# Analytics Modules for Business Students

**DOI:** 10.1007/s43069-023-00216-5

**Published:** 2023-05-01

**Authors:** Paula Carroll

**Affiliations:** School of Business, University College Dublin, Dublin, Ireland

**Keywords:** Business analytics, Teaching & learning, Analytics pedagogy, Analytics curriculum

## Abstract

Data science is a relatively new requirement for business students. Historically, many business students shied away from business statistics and quantitative or operational research (OR) modules believing them to be boring and irrelevant. The high-profile use of analytics and modelling during the COVID pandemic has drawn awareness to the relevance of analytics. Greater availability of data and modelling tools afford business students an opportunity to re-engage with operational research and analytics and to enjoy the satisfaction of modelling and solving real-world problems, but the challenge of the mathematical modelling skills gap of business students remains. In this paper, we describe a learning pathway of modules in business analytics that can enhance business students’ confidence and capabilities in performing statistical and analytical business tasks. We recommend modelling tools and incremental innovative mathematical modelling teaching approaches that are pedagogically sound and suitable for business students with varying quantitative backgrounds.

## Introduction

Davenport and Harris heralded the analytics age and highlighted the competitive advantage of companies that engage in quantitative, fact-based analysis [1]. The identity and branding of operational research (OR) has long been discussed, and boundaries between the quantitative disciplines related to analytics are blurred in practice [2, 3]. Computational techniques, analytical number crunching, and IT data management are more often the focus of the technical skills developed in data analytics programmes in Schools of Computer Science, Engineering, or Mathematics. In contrast, Schools of Business may highlight the need for soft skills in business understanding and communication, and aim to carve out a curriculum of complementary interdisciplinary analytics modules or programmes connecting analytics with with the more familiar traditional functional areas of business. Business Schools may offer programmes in “Business Intelligence” (BI) or “Business Analytics” (BA). BI generally tends to be associated with data management, reporting, and descriptive and diagnostic methods. BA is also associated with descriptive and diagnostic methods, but extends to predictive and prescriptive methods. These prescriptive methods may encompass the optimisation methods traditionally viewed as the core of the OR discipline.

For the purposes of this paper, we distinguish between data analytics computational techniques, and focus on BA taught in business schools which aims to extract actionable (business) insights from data. Functional areas of business have already harnessed opportunities in analytics with Accounting analytics [4, 5], Human Resource analytics [6, 7], and Marketing analytics [8, 9], emerging as sub-disciplines in their own right. The authors of these papers aim to provide definitions, clearer identities, research agendas, and education needs of these $X-$analytics sub-disciplines which include the development of mathematical modelling and implementation competences.

### Professional Developments in Analytics

Professional organisations have moved to provide continuous professional development programmes to allow their members keep their skills up to date. Professional accreditation demonstrates a commitment to a profession, and allows individuals to earn trust and gain credibility. Learned bodies and professional associations such as OR Societies play a role in developing a profession and the underlying discipline. They drive quality assurance goals and develop professional codes of ethics and behaviour, and technical competency standards.

INFORMS founded a Certified Analytics Professional (CAP) programme in 2013 which covers core elements of the analytics process. The UK OR Society provide pathways to a joint UK OR Society and UK Science Council Chartered and Registered Scientist, and to the INFORMS’ CAP accreditation.

The Analytics Institute of Ireland offers a professional certification which is mapped to the EDISON (Education for Data Intensive Science to Open New science frontiers) Data Science Framework. This framework is an EU initiative to bridge the digital skills gap and establish a data science profession.

### Academic Developments in Analytics

Turning to higher education and academic programmes, the disruptive impact of analytics needs on business education is discussed in [10]. The authors note the challenges to academia to keep pace with the fast paced technology industry and recommend innovation and the use of adaptable curricula that address multiple levels of data analyses. Innovation can be perceived as a process, or as a discrete item and can be disruptive or incremental. The importance of all types of innovation in creating and maintaining competencies is highlighted in [11]. However, innovation in education (pedagogic innovation) must be systemic, consistent, and scalable [12].

Academic accrediting organisations such as Association to Advance Collegiate Schools of Business (AACSB), the European Foundation for Management Development Quality Improvement System (EQUIS), and disciplinary accrediting societies such as the ACM and IEEE play a role in curriculum development. The AACSB note that analytics, data mining, data storage and reporting are key components of undergraduate degrees in business, and BA needs to be included to strengthen programs [13]. The 2018 accreditation guidelines expect business programmes to provide learning experiences in the application of statistical tools and techniques, data management, data analytics and information technology, and that business students should be able to analyse unstructured problems, formulate and solve them using appropriate technology [14].

In 2016 only a minority of 215 US colleges offered business analytics programs at varied academic levels [15]. By 2021 some 333 US colleges offered Masters in Business Intelligence and Analytics, with others offering mixed BA/MBA programmes. Similarly, business schools across the rest of the world now offer BA/BI programmes or include BA/BI modules in general business degree programmes. The intersection of the “Intelligence” and “Analytics” labelling is in itself interesting and indicative of the intersection of informatics, data science, and analytics, but also the challenges to establishing a clear identity of BA programmes. The boundaries between the BA and OR disciplines are blurred in a holistic view of the world according to [2].

### Analytics Modules for Business Students

We cannot talk about the innovative use of modelling languages and technical computing environments without understanding the baseline and target audience. Innovation at programme level fails if there is a lack of incremental innovation at the module level. Business students in general lack mathematical modelling competences and may struggle with mathematics symbolism. Providing business students exposure to a range of BA, BI and OR topics gives a systems level holistic view of the relevance and opportunities for analytics and OR to solve real world problems but requires us to address both the modelling skills gap and the mathematical language and symbolism challenge.

Educational innovation must match changing industry demands in business environments that are impacted by information technologies. Technology-enhanced learning innovation can increase the practical relevance of theory-driven curricula, but many universities and business schools struggle to integrate ICT efficiently into their programmes [16]. In this paper we focus on the use of modelling languages and technical computing environments in tandem with quantitative business module Learning Outcomes. The desired outcome of the innovation and modelling activities is to facilitate students to meet specific module learning outcomes. We articulate a pathway of modules with a series of incremental rather that disruptive innovations to using modelling languages and technical computing environments for general business students and BA graduate conversion students.

We describe a set of BA modules and teaching approaches that develop the analytics competencies of business students through gradual exposure to mathematical modelling concepts and their implementation through modelling languages. These modules are targeted at business students rather than engineering, computer science, mathematics or life science students who may need more computationally focused data analytics modules. We focus on academic modules rather than continuing professional development modules. We describe a pathway of BA modules leading from early stage undergraduate to graduate level. In particular we address the following research questions:

1. Which Learning Outcomes provide a foundational BA pathway for general business students?
2. Which pedagogical approaches and modelling tools are useful to incrementally innovate mathematical modelling teaching for business students?

We use the experiences in teaching business analytics at a business school in a large public university in Ireland as a case study to answer our research questions. The structure of the paper is as follows: Sect. 2 provides a literature review of the skills and knowledge to be developed in BA programmes, along with associated teaching and learning issues. Section 3 describes a pathway of BA modules for business students based on the experience of programmes at the College of Business at University College Dublin (UCD). Finally, discussion, conclusions and recommendations follow in Sects. 4 and 5.

## Business Analytics Educational Requirements

Academic institutions make incremental changes to appeal to a new audience and to respond to industry demands [17, 18]. Schools of Business historically included statistics and introductory OR modules, and have responded to the Analytics age by updating the quantitative modules of general business progammes, and by offering specialist programmes in BA.

Wilder and Ozgur (2015) propose five knowledge domains for a BA curriculum for undergraduate majors: project life cycle, data management, analytical techniques, deployment, and a functional area. The authors assume that students will have achieved some minimum level of proficiency with Microsoft Office before they begin their major studies [18].

The five knowledge domains are consistent with the skills required to implement the INFORMS seven step analytics process: 1. Business Problem Framing, 2. Analytics Problem Framing, 3. Data, 4. Methodology Selection, 5. Model Building, 6. Deployment, 7. Lifecycle Management.

A summary of the topics taught on Business Analytics programmes in the US in [19] highlights the broad multidisciplinary interest and variety of topics included. Topics receive different emphasis depending on the programme focus. We group and order the main topics to highlight the links between the topics and a typical value chain which progresses from data to understanding by processing the data, and using statistical modelling and analyses to create information that supports decision making. Further along the value chain optimisation models and simulation provide higher level support for decision analysis and decision making. We argue that the further along the value chain the greater the need for mathematical modelling language skills. The main BA topics are:

1. Data Handling (database, data warehousing, mining, BI);
2. Statistics (introductory, regression, multivariate analysis, forecasting/time series analysis, design of experiments 6-Sigma);
3. Modelling (OR/Management Science, risk);
4. Simulation;
5. Decision analysis (Management Information Systems, Decision Support Systems).

These topics and associated techniques align with the four types of analytics in the Gartner Analytic Ascendancy Model: descriptive (what happened?), diagnostic (why did it happen?), predictive (what is likely to happen?) and prescriptive (how can we make it happen?).

Academia helps to shape the discipline by covering specific topics and developing particular skill sets of professionals who then call themselves analytics specialists [19]. What is taught in university propagates into industry, while what is developed by the digital technology companies drives the agenda through the availability of analytics tools. Academic programme goals are achieved though the modules taught on the programme. Each module should state a set of learning outcomes: what a student will be able to do in some measurable way. A given competency may be associated with more than one measurable learning outcome. The module description should state which assessment instruments will be used. The module should be designed choosing assessment instruments that best measure the degree to which a student demonstrates their attainment of the learning outcome.

### Challenges to Teaching BA and OR to Business Students

Educators must not only know their own way around a discipline, but must know the conceptual barriers likely to hinder others. These conceptual barriers differ from discipline to discipline [20]. “Pedagogy” helps to locate our work within a tradition of thinking about learning and teaching, and to tailor how we teach to a target audience.

Initiatives to introduce quantitative topics to younger students are discussed in [21]. An early introduction can increase students’ interest and motivation towards OR and mathematics. The authors note that some of the mathematical skills required to approach OR are part of a standard mathematical background of secondary school pupils.

Undergraduate business students are generally required to meet minimum mathematical entry requirements but often demonstrate varied mathematical abilities and levels of understanding of mathematical concepts. Business students consider quantitative courses the most difficult and challenging courses in the business curricula [22, 23].

Mathematical meaning and understanding derives from embodied cognition [24]. The use of symbols to convey ideas and understanding, using mathematical concepts as the building blocks and set of rules to generate understanding pose a challenge to many business students. Embodied cognition researchers explore how mathematical concepts are experiences in visual and sensory motor memories. They note the ways we posture, gaze, gesture, point, and use tools when expressing mathematical ideas as evidence of our holding mathematical ideas in the motor and perceptual areas of the brain. For example we count on our fingers as children, but use the same process as adults to mentally count on our fingers. Children learn the count sequence by rote before understanding the numerical meaning of number words and Arabic numerals [25].

Numerical symbol knowledge encompasses multiple aspects of symbolic understanding. Maths and analytics education emphasise number sense but cannot be disconnected from the associated symbol sense [26]. The linguistic challenges of mathematics are discussed in [27] who highlight the importance of teachers talking to apprentice students in the technical language of mathematics. This way of speaking allows students to construct complex meaning relationships in the problems students have to solve.

Business students have real difficulties transforming verbal reasoning (a real-world natural language representation) into algebraic symbols and vice versa. Mathematical and statistical symbols and language are required to encode the real world problem into a format that can be solved by computer (algorithm) [28]. This emphasises the importance of Schleppergrell’s advice in [27] to use the technical language of mathematics to convey meaning to build students’ mental models of relational processes. While recognising that the technical vocabulary is a challenge, particularly for business students, it is the basis of grammatical patterning than can be transformed into the correct syntax of a problem formulation. The innovative use of modelling languages may fail if the target audience, module learning outcomes and innovations are not well matched. Innovations in teaching OR and Integer Programming using technical vocabulary in a way that motivates and engages business students are described in [29–32].

### Frameworks for Teaching BA and OR

When industries first emerge, they are highly dependent upon intuitive experts who can operate on heuristic rules [10]. For analytics to become sustainable, the intuition needs to be replaced with a clear process map. We aim to provide students a framework to guide them through the business analytics process.

Analytics as a data science makes use of the Cross-Industry Standard Process for Data Mining (CRISP-DM) Framework [33]. CRISP-DM has six steps (Business understanding, data understanding, Data Preparation, Modelling, Evaluation and ends with Deployment. The INFORMS CAP seven steps of analytics projects are likely to be familiar with BA and OR academics: (1) business problem framing, (2) analytics problem framing, (3) data, (4) methodology selection, (5) model building, (6) deployment, (7) lifecycle management. The final lifecycle step closes the loop—an important consideration in project management and sustainability.

Steps 1 and 2 of the INFORMS analytics process (Business, and Analytics Problem Framing) are a challenge for business students. The linguistic challenges noted in Sect. 2.1 play a role. There is a bigger gap for students to close since there is more scope for creativity in the problem structuring. Providing examples of formulated problems and practice problem sheets helps students in this regard.

A problem-solving framework to teach introductory statistics to business students in [34] recommends following five steps: define, collect, organize, visualize, analyze. Teachers of OR/Management Science will have used a similar framework to steer students toward some problem structuring method to assist students understand how to map a real world problem to a manageable problem statement, and toward a mathematical formulation and useful model implementation to solve the problem.

There are also useful transferable ideas in the Guidelines for Assessment and Instruction in Statistics Education (GAISE) [35] which advise that demonstrating theoretical principles on real world data with real use cases helps students make connections with the underlying statistical concepts.

In the case of optimisation, business students often have difficulty translating a verbal description of a business problem to a mathematical programming formulation, the algebraic symbols sometimes getting in their way rather than supporting the transformation. A quote in [36] is worth noting: “In school the professor formulates the [mathematical] problem and you solve it-you hope. In industry, you formulate the [mathematical] problem and the software solves it-you hope”. This highlights the modelling and mathematical language competence gap.

Once formulated, the model building step is more straightforward. Although students still need advice on the language syntax, sensible test and debug, and the final life cycle management step of the INFORMS process: archiving with systematic file naming conventions.

The final challenges for students are the solution interpretation and communicating a decision recommendation to decision makers in the INFORMS Deployment step. The need for “story telling” to avoid a breakdown over the “last mile” of analytics and connect the decision maker to the outputs is highlighted in [37].

The theoretical principles of the topics covered on any BA programme are independent of the software and tools. Modern software tools are widely available as open source or via academic partner programmes. Most have user-friendly interfaces which means topics can generally be demonstrated, eliminating the need for tedious error-prone hand calculations. As noted in [38], mathematically most optimisation is not difficult, but writing efficient, reliable, scaleable, error-free code is, and is beyond the capability of general business students. While the use of software tools has the benefit that “modestly talented people can accomplish increasingly sophisticated tasks” [10], it may hinder understanding if used as a pure black-box tool. There is a balance to be struck, and solving small examples visually or “by hand” still plays a role to aid student understanding of the underlying principles and algorithmic approaches.

## Business Analytics Developments at University College Dublin

In this section, we outline BA modules that are offered at the UCD College of Business as part of general and specialist undergraduate and postgraduate programmes. We use the experiences in teaching business analytics at UCD to demonstrate a case study of suitable Learning Outcomes and teaching approaches to answer our research questions.

The UCD School of Business is accredited by both the AACSB and the EFMD (European Foundation for Management Development) EFMD Quality Improvement System (EQUIS). The School offers general undergraduate business degree programmes (European qualifications framework level 8).

An undergraduate BSc in Business Analytics was launched in 2015 but this innovation failed to attract sufficient student numbers and was cancelled in 2018. It seems we were not able to persuade students of the opportunities in the emerging BA discipline. Ireland does not have a strong history of heavy industry and the use of OR, but is the home to the European headquarters of multinational digital technology companies such as Google, Facebook, Microsoft, and E-bay, and still the identity and branding of BA and OR remain a challenge to communicate to undergraduate students and the general public in Ireland. The business analytics competence gap remains a challenge at undergraduate level.

UCD launched an MSc in Business Analytics in 2008 which is ranked 23rd in the 2021 QS World University Rankings. There is high demand with 120 students taking the programme online in 2020/2021 due to COVID public health restrictions. The high demand and success of this innovation is in part fed by members of the workforce upskilling after the 2008 financial crash as noted in [37], and the need for business-related digital skills by non-business graduates from engineering and quantitative backgrounds. This makes for a diverse group of students with a broad range of cultural and disciplinary backgrounds, and work experiences. Numbers were capped at 150 in academic year 2021/2022. Taking student numbers as a measure of success of business analytics programmatic innovation, the undergraduate programme failed and the MSc conversion graduate succeeded.

In the next section, we describe a series of modules taught by the author that form part of a pathway for undergraduate business students to the graduate MSc BA programme. We also describe a core optimisation module and an elective in Operational Research on the MSc BA programme.

Table 1 gives a brief summary of the BA modules discussed in this paper. Left to right shows the module code, name, which stage they are taken at, whether they are core or elective, and the average number of students taking the module per year.

**Table 1:** Sample BA and OR modules in the UCD College of Business

Code	Name	Stage	Status	*n*/year
MIS10090	Data Analysis for Decision Makers	1	core	600
MIS10060	Introduction to Business Analytics	1	core	75
MIS30040	Analytics Modelling	3	elective	75
MIS30140	Business Analytics Project	3	elective	15
MIS41160	Optimisation for Business	4	core	120
MIS41090	Advanced Operations Research	4	elective	50

**Fig. 1:**
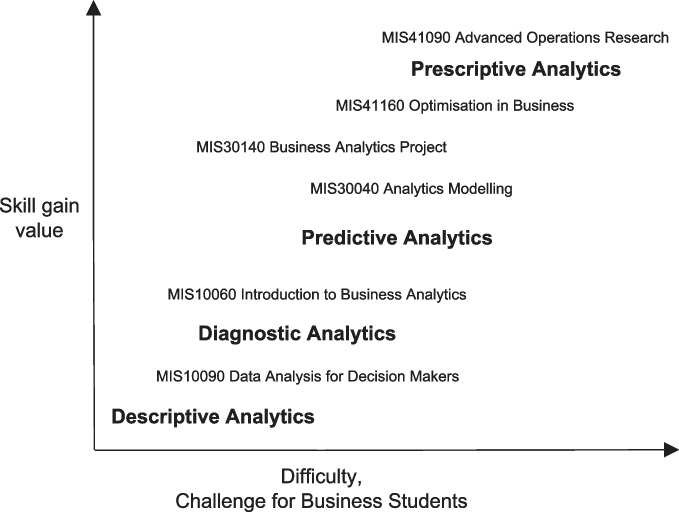
Business Analytics Modules at UCD

Figure 1 shows the relation between the topics covered by the modules in a typical analytics ascendancy model, progressing through a Descriptive - Diagnostic - Predictive - Prescriptive analytics taxonomy. The positioning on the difficulty/challenge for business students is subjective based on the author’s 20 years of teaching experience.

### Teaching Approach, Learning Resources, and Assessment

Nobel laureate Herb Simon said “Learning takes place in the minds of students and nowhere else, and the effectiveness of teachers lies in what they can induce students to do. The beginning of the design of any educational procedure is dreaming up experiences for students: things that we want students to do because these are the activities that will help them to learn this kind of information and skill” [39].

The author’s observations of business students trying to learn validates Simon’s claim. Business students learn best by example and experimentation. The linguistic challenges noted in Sect. 2.1 play a role here. In the same way that we learn to understand spoken language before we can speak it fluently, students can become proficient at reading and understanding the algebraic notation of formulated problems by seeing examples, and then become more familiar with problem formulation through the use of practice exercises. This incremental approach is a constructionist approach. Following the advice of the GAISE guidelines [35], we use a blended learning approach with lecture materials made available on the Virtual Learning Environment (VLE).

For early-stage students, we create short videos and devise step-by-step tutorial tasks and practice exercises. The videos aim to improve students’ grasp of the use of symbols in statistical concepts, and to improve statistical literacy by assigning meaning to symbolic algebraic statements. Students need to be able to recognise the relationship between the things of the mathematical grammar and the processes of the mathematics reasoning [27].

Mathematics symbolism has been developed to express mathematical concepts. The algebraic symbols and notation of a mathematical programming formulation are part of an international language conveying meaning to a community of fluent writers and readers. The videos aim to help students understand mathematical symbols and notation, and so build toward creating their own mental models of more complex mathematical relationships. The videos were developed using a mixture of Articulate, Captivate and with the support of a research assistant—Adobe Illustrator and AfterEffects. We note that there is a steep learning curve in multimedia content development, and that often a low-tech approach such as powerpoint animation can be quite effective. Further reflections on the learning content development are given in [40].

For graduate-level students, we recommend standard textbooks such as [41, 42], and curate a selection of ebook chapters and short journal articles on specific topics which are available in the VLE and from the university library.

All modules except MIS30140 are assessed through a combination of continuous assessment and traditional end of semester written exam. Openbook online multiple choice quizzes and a team project are the basis of the formative assessment, with a combination of theoretical and practical exercises forming the summative end of semester exam. The team project is designed to synthesis the topics of each module and tasks the team with modelling and solving BA or OR problems. The team is required to write a short report explaining their problem, model, solution approach, and recommendations. Most business students have excellent communication skills and seem to enjoy this “story telling” element of the coursework. The projects are tailored to the stage of the students. More details are given in [30].

MIS30140 takes an enquiry-based learning (EBL) approach. Students work individually to frame and scope a BA or OR problem during the EBL contact sessions. Data sources and solution approaches are explored with the student deciding and justifying their final choice. There is no final exam with 100% of the marks awarded for three continuous assessment components: a management report, the code and data used to solve the problem, and an oral presentation on their project.

In the next section, we describe the BA modules and their learning outcomes in more detail.

### Modules for Early-Stage Business Students

All general business students at UCD are required to take an introductory course in statistics. The average age of the students at the start of the academic year is 19.1 years with 41% female, 59% male, although the intake is becoming more gender balanced in recent years. The majority are Irish nationals ($\sim 80\%$) having completed their high school education in Ireland and having completed the Irish state leaving certificate exam. The state exam allows students to take a number of core subjects (English, Irish, and Mathematics), along with a choice from a wide range of subject. Students may sit the exams at two different levels: a higher level which covers more advanced topics in some depth, and ordinary level which has a narrower focus and less breadth. UCD business programmes stipulate minimum entry requirements in mathematics. Broadly speaking, students who take the higher level Irish leaving certificate exam have demonstrated higher levels of mathematics ability with $\sim 70\%$ of business students having taken higher level mathematics.

The Learning Outcomes (LOs) for the core MIS10090 Data Analysis for Decision Makers are:

1. Prepare spreadsheet models to store, manipulate and analyse quantitative data using common probability distributions and statistical functions;
2. Calculate, analyse and present useful statistical measurements from large-scale datasets;
3. Create and interpret inferential statistical statements about population parameters;
4. Interpret the results of data analyses with a view to informing decision making.

Descriptive analytics is at the bottom left of Fig. 1 in terms of difficulty and skill value, but exploratory data analysis (EDA) is a critical part of the Data step in BA projects. Students are familiar from their high school studies with the statistical moments such as average and variance but have most likely used a calculator to calculate results on small datasets rather than a computing environment. Microsoft Excel is an ideal tool to introduce business students to the ideas of data types, structured data and syntax, and to extend their capabilities to explore larger datasets. Schleppegrell (2007) notes that mathematics is highly technical, with characteristic patterns of vocabulary and grammar [27]. Words used in everyday language such as “sum” and “average” have an intuitive representation in MS Excel, yet students have to get the syntax right to get a correct result. This is a gentle first introduction to modelling languages.

General business students take an Introductions to Business Analytics module. The LOs for MIS10060 Introduction to Business Analytics are:

1. Identify the main classes of analytics problems arising in business and industry;
2. Formulate, explain, and distinguish between various forms of regression, classification, and linear programming models;
3. Create and implement regression, classification, and linear programming models (in suitable software);
4. Test the models and interpret results in a form suitable for a business client or manager.

We use Microsoft Excel again as students now have some familiarity with its functionality can support simple and multiple linear regression and linear programming. With some manipulation, students can also build logistic regression models in Excel.

A meaningful link can be made between the ideas of summation and linearity in linear regression models, which can then be expanded on in explaining the linearity of Linear Programming objective functions and constraints. At this point it is useful to draw students attention to the mathematical linguistic vocabulary term “sum”, and the corresponding algebraic notation as shown in Eq. 1.

1 $$\begin{aligned} y = \beta _0 + \beta _1 x_1 + \beta _2x_2 +....+ \beta _mx_m\rightarrow y = \sum _{i \,=\, 0}^m \beta _ix_i \end{aligned}$$

The Greek sigma symbol for summation appears on the MS Excel toolbar. During tutorials, students practice implementing linear regression and simple explicit LP formulations with a small number of decision variables and constraints in Microsoft Excel.

### Elective Models for Final-Stage Business Students

In their final year, business students at UCD can choose elective modules and focus on a speciality like Accounting, Banking and Finance, Business Analytics, Marketing, Human Resources, etc. Students with more interest in quantitative methods can self-select from a range of modules offered by the MIS subject area including an introduction to programming, which currently uses Python to write programs to perform logical analysis and processing of business data.

LOs from two elective modules that form a BA pathway are shown below.

MIS30040: Analytics Modelling

1. Discuss a portfolio of important business optimisation problems and their application;
2. Explain the concepts of a suite of key linear programming and network approaches;
3. Formulate appropriate mathematical models of real world business optimisation problems;
4. Implement and solve the mathematical models using suitable computer packages such as FICO XpressMP and Microsoft Excel;
5. Demonstrate how to aid business decision making by interpreting the solutions to recommend decisions.

MIS30140: Business Analytics Project Learning Outcomes:

1. Demonstrate a capacity to identify a business analytics problem and relate it to problems described in the literature;
2. Specify a business analytics problem in a mathematical/statistical modelling framework;
3. Select and implement an appropriate business analytics methodology to solve the problem;
4. Analyse and present the results of the solution with justifiable recommendations.

Having made the case for using MS Excel to introduce general business students to analytics, we note the challenge of controlling a master copy of user-developed spreadsheets, and of debugging them with an estimated 20% - 40% of spreadsheets containing errors [1]. The badly thought-out use of Excel in England during the second wave of the Covid pandemic was the reason nearly 16,000 coronavirus cases went unreported. Information about confirmed cases was truncated as Public Health England used an older version of Excel which limited the number of rows. It took about two weeks before this simple data processing error was recognised and corrected.

Since the final-stage students have self-selected these elective modules, they tend to be more self-directed and focused and have an aptitude for quantitative approaches. At this stage of their studies, these business students are more comfortable with mathematics symbolism and can move toward converting a natural language description of a problem to an algebraic representation. Integer Programming is introduced as an extension of LP. Small IP examples are implemented in Excel, and students are introduced to the ideas of scaleability and tractability.

UCD participates in the FICO XpressMP Academic Partner Programme [43], and students are introduced to the Mosel modelling language and the XpressMP ILP solvers.

For many business students, this is their first introduction to a programming language and interactive development environment, although some may also be taking the introduction to programming elective. Explicit formulations of small ILP problems are coded in Mosel in practice exercises. See sample “pottery” Mosel implementations of a small two decision variable, two-constraint product mix problem based on an example in [42].

The idea of using the Mosel modelling language constructs to map from explicit expressions to an algebraic formulation to implementation in a modelling language is the next subject of several practice exercises. Equation 2 shows a sample ILP objective function.

2 $$\begin{aligned} z = c_1 x_1 + c_2x_2 +....+ c_mx_m\rightarrow z = \sum _{i \,=\, 1}^m c_ix_i \end{aligned}$$

Equation 2 can be implemented in Mosel as: z:= sum (i in 1..m) c(i)*x(i)

This learning approach highlights the links between the syntax of a programming (modelling) language and the grammar of a mathematical expression. It also demonstrates the idea of data types, assignment and the concepts of arrays, and indexing. The practice exercises gradually introduce these ideas incrementally adding code snippets to progress from hard-coded explicit LP models up to the level of separating the parameter data from the problem instance, and using Mosel language keywords such as “sum” and “forall” to implement the algebraic formulation of a MILP.

Lastly, the ideas of function signatures and library calls are highlighted by calling the Mosel library maximise or minimise functions to solve the LP. While Mosel can be used as a programming language, we only use it to implement and solve MILP models.

### Models for Graduate Conversion Business Students

In this final section, we describe two of the modules delivered as part of the MSc Business Analytics programme. This is a 1-year full-time or 2-year part-time programme. The students are a diverse group, the 2020 cohort had a split of 43%/57% Females/Males, represented 15 nationalities, and had an average age of 25 years. Many have several years of work experience in data engineering perhaps using a “low-code/no-code approach”, and may not have a formal business qualification. Some are general business students aiming to develop depth in BA. Core modules include programming (Python in recent years), statistics, and optimisation. Learning Outcomes for the core optimisation module in the first trimester and an elective OR module in the second trimester are shown below:

MIS41140: Optimisation in Business

1. Translate a verbal optimisation problem into a formal mathematical model and select a computational solution approach;
2. Choose an appropriate software tool to implement and solve the mathematical model;
3. Interpret the solution results in detail and discuss the limitations of the chosen solution approach;
4. Describe the similarities and differences between different optimisation approaches.

MIS41090: Advanced Operations Research

1. Define convexity in optimisation, with particular reference to the special cases of Linear, Integer Linear, and Quadratic Programming;
2. Formulate a problem given a verbal description and decide whether it is convex;
3. Translate problems into the language of a standard software solver and execute it, and interpret the results;
4. Understand special cases of routing and network problems as they arise;
5. Execute standard algorithms for network partition, flow, and others;
6. Describe applications of network models in analytics.

Students at this level are more mature and manage their own learning well. They tend to be more pro-active in engaging with the designed educational experiences. Again a set of practice exercises aim to assist students to move through ideas understanding scaleability and tractability.

**Fig. 2:**
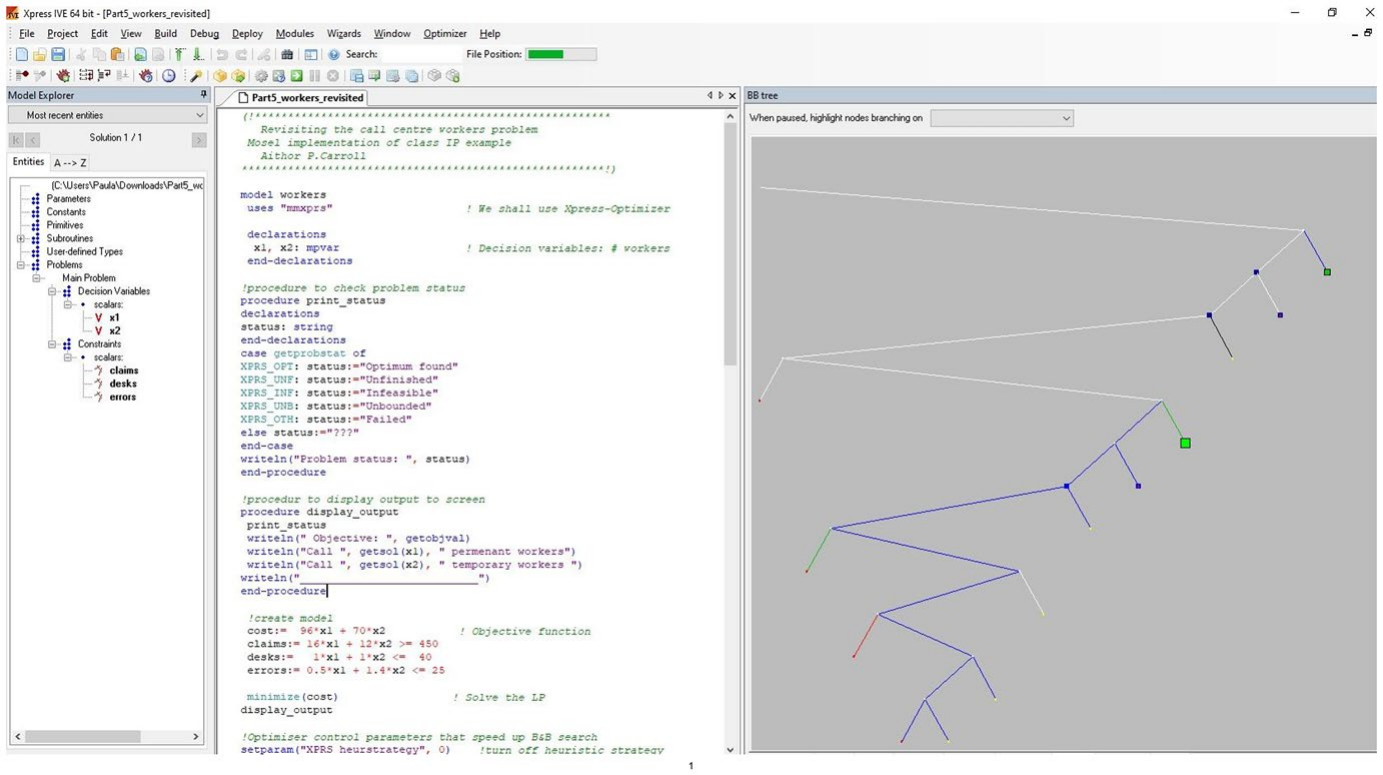
Branch and Bound Tree Development

Hands-on exercises building and visualising branch and bound trees in Mosel allow for discussions during class on effective algorithms and hyperparameter design choices. Figure 2 shows a screenshot of a useful small IP problem which is solved efficiently by the XpressMP IP solver. Turning off the default heuristics exposes a much larger branch-and-bound tree that would have had to be explored otherwise.

In other exercises, students are asked to compare the implementation of sample models in Microsoft Excel and XpressMP Mosel. Students are asked to implement a shortest path model and comment on the model development process in each case, and the re-usability and test-ability of their models. Students are also given sample problems ranging from small “toy” examples to larger more realistic problems. They have to write a report to explain and justify their modelling choices and assumptions. Discussions during class exercises point out that all models are wrong in some sense, and we are restricted to translating the real-world problem to a tractable useful version. Students become aware of their model limitations by testing on different or larger problem instances.

## Discussion and Analysis

Returning to our two research questions, the modules described support the development of competencies and skills that in aggregate span the analytics ascendancy from descriptive through to prescriptive techniques, and enable students to perform the tasks in the INFORM seven step analytics project model. The LOs associated with the modules specify which BA competencies are measured. We discuss how the LOs provide a foundational BA pathway for general business students (research question 1), and discuss the usefulness of our pedagogical approach (research question 2).

### BA Pathway of Learning Outcomes

In aggregate the modules described in Sect. 3 develop the skills and competencies to implement the INFORMS seven steps of an analytics project. The learning outcomes express the BA tasks a business student will be able to do as measurable outcomes for specific competencies in business analytics. The students develop basic proficiency in performing analytics tasks in the early-stage modules. Beginning with data analysis (MIS10090), the learning outcomes specify descriptive, diagnostic tasks associated with traditional statistics. Students’ ability to perform the tasks are measured by the openbook MCQ, team assignment, and end of semester exam assessment instruments. This covers descriptive and diagnostics analytics in the analytics ascendancy framework.

Early-stage students meet predictive and prescriptive analytics at an introductory level in the Introduction to Business Analytics module (MIS10060). The module learning outcomes specify tasks in regression, classification and LP model building. Similar assessment instruments are used as in the data analysis module.

Moving along the analytics value chain, the higher level of difficulty of prescriptive optimisation tasks requires a deeper knowledge of problem formulation and implementation tools. Later stage undergraduates can choose the elective modules (MIS30040 or MIS30140) to deepen their knowledge of prescriptive analytics techniques and develop a higher level of competency in formulating and implementing optimisation models. The Business Analytics Project module gives students an opportunity to explore the seven steps of the INFORMS analytics project with freedom to focus on any of the steps in more detail. The project module uses a 100% continuous assessment approach while the analytics modelling module uses the more traditional MCQ, team assignment, and end of semester exam assessment instruments. The students progress from a basic to a more capable competency level on successful completion of these modules.

Finally the graduate modules focus on prescriptive analytics, the highest value component of the ascendancy hierarchy. While some of the topics covered are similar to the undergraduate offerings, the students are expected to reach a more accomplished competency level. The MCQ, team assignment and end of semester exam assessment instruments are modified accordingly to assess for evidence of deeper understanding and modelling ability.

### Pedagogical Approaches and Modelling Tools for Business Students

The modules provide opportunities for business students to gain BA domain knowledge and to develop analtyics behaviours. Successful students will have developed analytics competencies and applied skills and knowledge to successfully perform the steps of the INFORMS analytics project.

Our experience in working with general business students resonates with the published literature, that business students can perform sound technical analysis but may not be interested in the area and often struggle to extend their learning to unstructured real world situations. The learning resources and experiences aim to develop students’ confidence and support progressive levels of mastery in implementing analytics tasks. We assess whether the incremental innovations in teaching mathematical modelling and language had the desired outcomes and supported the student to attain the module LOs. Early-stage students generally enjoy the practical side of EDA using Excel (MIS10090 Data Analysis - LOs 1 and 2). However, they find the theoretical principles of inferential statics and probability more challenging (LOs 3 and 4). Failure rates remain stubbornly high at $\sim 17\%$ despite the attempts to induce the students to engage in educational experiences and influence what the student does to learn. However, $\sim 95\%$ of those students pass on their second attempt. A further detailed discussion of what business students do with the learning resources appears in [22]. The pass rate on the elective undergraduate, and postgraduate modules is consistently 100%. Student feedback shows that students enjoy the hands-on exercises and they comment on how this helps them align the theory to the practice. Drop out rates across the programmes are low with high student satisfaction rates.

## Conclusion and Recommendations

The topics and teaching approach on BA and general business programmes vary depending on the student cohort. Spreadsheets remain an important tool for general business students. Microsoft is still a dominant force with widely available and a widely used suite of tools (Gartner magic quadrant 2021). Final year and graduate conversion students can be introduced to more formal modelling approaches and commercial software such as FICO XpressMP. Individual instructors influence student learning outcomes and enthusiasm for the BA and OR disciplines. Our paper has shown examples of modules with learning objectives and experiences to support general business students on an analytics pathway.

There is high demand for business students with a mix of technical and softer business skills. There is still work to be done to communicate the opportunities of business analytics to early-stage students. This is an important consideration in building a pipeline of future skilled business graduates.

To maintain the pipeline, students need to be offered suitable modules and incremental mathematical modelling educational experiences. Developing high-quality educational experiences is resource intensive, and investment by academics in learning resource developments needs to be supported and rewarded in business schools.

There are many opportunities for further work in this area. In this study analytics is framed as requiring mathematical linguistic and symbolic understanding which requires an incremental innovation approach to the way we teach mathematical modelling to business students. There are challenges for students and educators to understand the underlying grammar and structure of the native language representation of problems. In the work to date, problems are framed by native English language speakers which may pose cultural and linguistic challenges for an international student body.

Finally, we note that BA specialists bring significant value by framing problems in such a way as to leverage available data and harness computational power. This art is unlikely to be automated and there are good career prospects for students of business analytics who learn the power of mathematical modelling.

## Data Availability

Not applicable.
